# A life cycle and product type based estimator for quantifying the carbon stored in wood products

**DOI:** 10.1186/s13021-022-00220-y

**Published:** 2023-01-16

**Authors:** Xinyuan Wei, Jianheng Zhao, Daniel J. Hayes, Adam Daigneault, He Zhu

**Affiliations:** 1 Environmental Sciences Division, Oak Ridge National Laboratory, Oak Ridge, TN 37830 USA; 2 Center for Research on Sustainable Forests, University of Maine, Orono, ME 04469 USA; 3 School of Forest Resources, University of Maine, Orono, ME 04469 USA; 4 Institute of Geographic Sciences and Natural Resources Research, Chinese Academy of Sciences, Beijing, 100101 China

**Keywords:** Carbon pool, Carbon storage, Estimator, Life cycle, Recycle, Wood products

## Abstract

**Background:**

Timber harvesting and industrial wood processing laterally transfer the carbon stored in forest sectors to wood products creating a wood products carbon pool. The carbon stored in wood products is allocated to end-use wood products (e.g., paper, furniture), landfill, and charcoal. Wood products can store substantial amounts of carbon and contribute to the mitigation of greenhouse effects. Therefore, accurate accounts for the size of wood products carbon pools for different regions are essential to estimating the land-atmosphere carbon exchange by using the bottom-up approach of carbon stock change.

**Results:**

To quantify the carbon stored in wood products, we developed a state-of-the-art estimator (Wood Products Carbon Storage Estimator, WPsCS Estimator) that includes the wood products disposal, recycling, and waste wood decomposition processes. The wood products carbon pool in this estimator has three subpools: (1) end-use wood products, (2) landfill, and (3) charcoal carbon. In addition, it has a user-friendly interface, which can be used to easily parameterize and calibrate an estimation. To evaluate its performance, we applied this estimator to account for the carbon stored in wood products made from the timber harvested in Maine, USA, and the carbon storage of wood products consumed in the United States.

**Conclusion:**

The WPsCS Estimator can efficiently and easily quantify the carbon stored in harvested wood products for a given region over a specific period, which was demonstrated with two illustrative examples. In addition, WPsCS Estimator has a user-friendly interface, and all parameters can be easily modified.

**Supplementary Information:**

The online version contains supplementary material available at 10.1186/s13021-022-00220-y.

## Background

Accounting for the carbon stored in harvested wood products is necessary to analyze the full function of forest ecosystems in sequestering atmospheric carbon and mitigating the greenhouse effect [1, 2]. In general, the carbon budget of end-use wood products pools is calculated as the difference between inputs from harvest and losses to decay or trade over a given period. Where inputs exceed losses over this period, carbon accumulates in wood products pools and represents a net sink of atmospheric carbon. Johnston and Radeloff [3] found that the carbon sequestered in end-use wood products served as a net sink of 90 Tg C globally in 2015. Zhang et al. [4] reported a larger carbon sink in global end-use wood products, with an average of 122 Tg C per year during the period of 1992–2015. This annual sink of harvested carbon is heavily influenced by demand and supply in the products market, which is impacted by various social-economic factors such as population and household income, technological advancement in the wood industry, climate and other environmental factors, and forest management strategies [5–8]. Therefore, an accurate accounting for the carbon stored in wood products is essential to assessing land-atmosphere carbon exchange by developing carbon budgets at regional, continental, and global scales.

The Intergovernmental Panel on Climate Change (IPCC) provides calculation guidance for estimating the size of harvested wood products carbon pools and their annual stock changes in three tiers of approach that can be used based on the availability of wood products data and the level of aggregation in the pool category definitions [9]. This guidance has been widely used to develop a considerable number of harvested wood products carbon accounting models and frameworks, which have been widely applied to various system boundaries [e.g., 10,11,12]. Brunet-Navarro et al. [13] reviewed 41 wood product carbon accounting models and summarized their characteristics. These models are different in their system boundary, spin-up, bucking allocation method, number of carbon pools, treatment of wood product disposal and recycling processes, as well as technological advancement in the wood industry.

The 2006 IPCC guidelines describe four approaches to define system boundaries for wood products carbon storage estimation [13, 14]. The stock-change approach estimates the carbon in wood products consumed and physically stored in the study area. The atmospheric flow approach estimates the carbon stored in wood products made from the harvested timber from local forests along with the emissions from wood products consumed in the study area, but the carbon emissions from the products exported to and consumed in other regions are not counted. The production approach estimates the carbon stored in wood products made from timber harvested in the study area. The carbon stock and emission from exported products are counted, but the carbon stock in imported wood products is not included in the calculation. Finally, the simple decay approach estimates carbon stored in wood products consumed in the study area. Meanwhile, the carbon stock and emission made from local forests but exported and consumed in other regions are also counted in this approach.

Wood products estimation models often use a “spin-up” process to account for the initial size of the carbon pool at the start of the period for reporting. The initialization is not always included in the accounting [e.g., 15], but the study or reporting time period should be well documented. Another strategy is to run the spin-up for a long enough period using historical wood production data to reach the equilibrium stage [16]. If the harvested timber is not adequately categorized into intermediate and end-use wood products, a bucking allocation process is required, which refers to the allocation of harvested timber to different wood products pools [17]. A carbon pool is typically defined as a group of wood products that have a similar life cycle [18]. Wood product disposal is the time point when products are retired from use and disposed [13]. The recycling process includes the waste wood material reused to make new products or to generate energy at the end of its service life [19]. Technological advancement in the wood industry may result in more carbon from the forest sector ending up in the wood products by reduce processing residuals, extended service life of each end-use wood product, and an increase in the recycling rate, which can significantly expand the wood products carbon pool size [20].

To estimate the size of a wood products carbon pool and its interannual stock changes, monitoring carbon inflow and outflow rates is the most popular approach [21]. Carbon in harvested timber initially flows into the overall wood products pool, and then allocated among the different products such as construction material, furniture, paper, and biofuel [22]. The end-use wood product is disposed of when it reaches the end of its service life. A part of the disposed wood products will be recycled to make new products or directly burned as biofuel to generate energy, and the remainder will be disposed to landfills. Waste wood materials in landfills will be slowly decomposed and the carbon released to the atmosphere. Therefore, using the life cycle of each wood product is an efficient method to realize the estimation of wood products carbon pool size over time.

In this study, we developed a life cycle and product type based estimator for quantifying the amount and interannual change in wood products carbon storage using the annual production of each product type, a service life based disposal method, a time-dependent recycling process, and a time-dependent decomposition approach for waste wood materials in landfills. To evaluate the performance of this estimator, we applied it to (1) account for the carbon storage in wood products produced by timber harvested in Maine, USA from 1961 to 2019 (system boundary: production approach) and (2) estimate the carbon storage in wood products consumed in the United States over the period of 1961–2020 (system boundary: stock-change approach).

## Methods

### Wood products carbon storage estimator

**Fig. 1:**
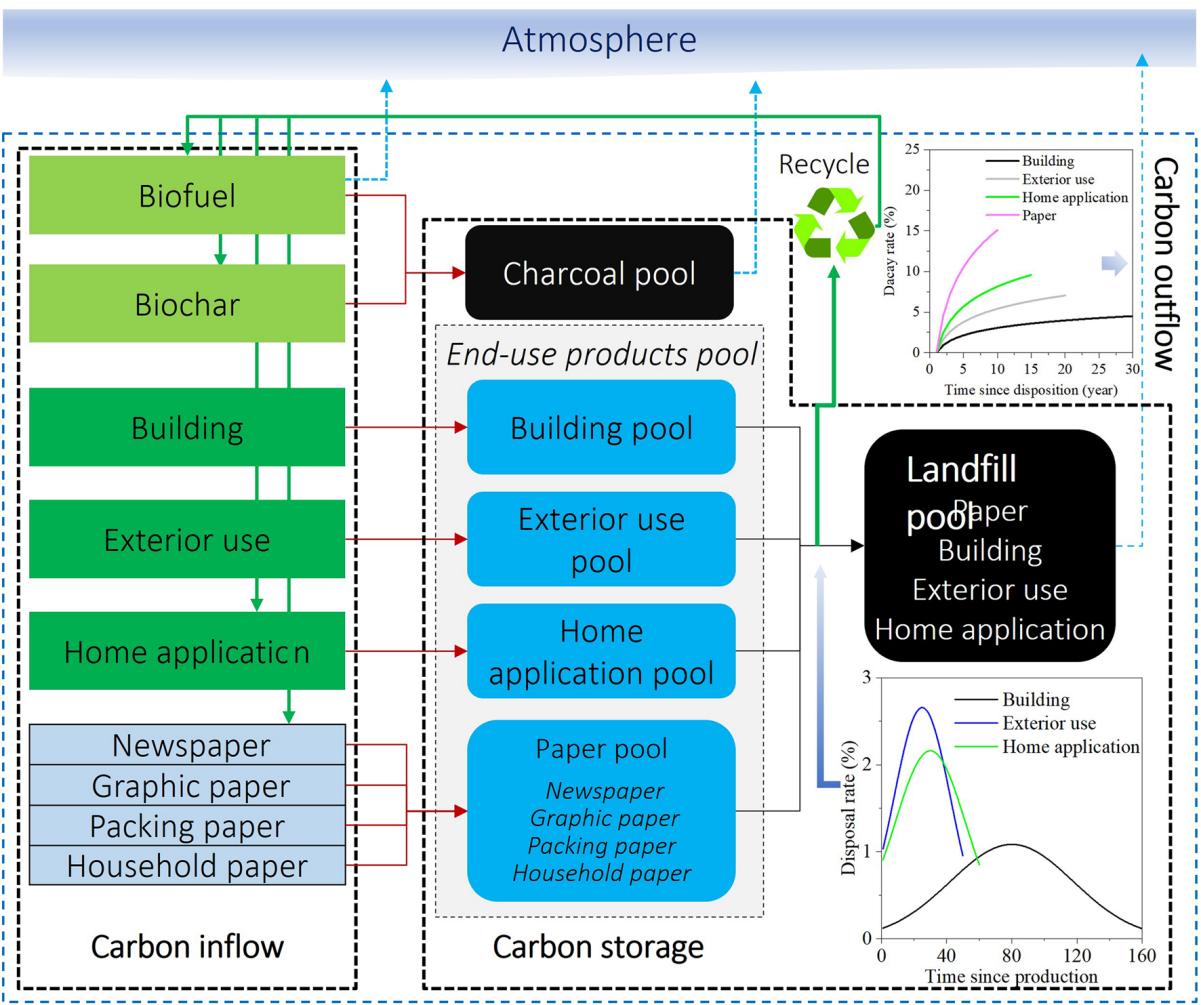
The structure, carbon pools, and carbon flux processes in the Wood Products Carbon Storage Estimator (WPsCS Estimator). Note that the biochar is non-energy use biochar

To account for the carbon stored in wood products including end-use products (e.g., building, furniture, and paper), charcoal, and waste wood materials in landfills, we developed the Wood Products Carbon Storage Estimator (WPsCS Estimator). WPsCS Estimator is operated at an annual time scale. The input data to run the Estimator consists of the annual consumption or production of wood product types within the user-defined system boundary including bioenergy, biochar, paper products (i.e., newspaper, graphic paper, packing paper, and household paper), building, exterior use, and home application. These wood product types are aggregated in three carbon pools: charcoal, end-use products, and landfill carbon (Fig. 1). According to the similarity of service life for different end-use wood products, the end-use wood products carbon pool is categorized to four subpools (i.e., paper, building, exterior-use, and home application), and the paper carbon subpool is further classified into newspaper, graphic paper, packing paper, and household paper. Finally, the landfill carbon pool is accounted for using four subpools: waste paper, building, exterior use, and home application materials carbon pools.

In WPsCS Estimator, the annual carbon input to the charcoal carbon pool consists of the production of non-energy use biochar and the charcoal formed by biofuel combustion (Fig. 1). Biofuel combustion directly releases most of the carbon to the atmosphere while, at the same time, a small portion of biomass is thermochemically converted to recalcitrant charcoal. To estimate the charcoal created by biofuel burning, a combustion efficiency is employed in the estimator. The combustion efficiency represents the portion of biofuel that is completely burned, and the remainder is converted to charcoal. Because the four paper products in the paper carbon pool are significantly different in their service lives, they are accounted for separately in the estimator. The building subpool stores carbon in wood products used in construction. The exterior-use carbon pool represents the wood products that are employed out-of-doors such as wood dock and railway tie. The home application carbon pool includes wood products used inside such as furniture and wood floor. Each of these subpools is assigned a service life in the estimator, and when the end-use wood product reaches the end of its service life, it will be disposed (Fig. 1). The disposed wood product can be recycled to create new products, used as biomass fuel, or directly disposed to landfills. Waste wood products in landfills will be decomposed and the carbon is eventually released to the atmosphere after a decaying period.

### Wood products carbon flux

Because charcoal is chemically and biologically stable, it has a relatively long residence time in the environment [23]. Therefore, although the annual production of non-energy use biochar and charcoal formed by biofuel burning is relatively small, the magnitude of charcoal carbon pool represents a potentially significant long-term sink of atmospheric CO_2_ [24]. The carbon stored in the charcoal pool can be released to the atmosphere by recombustion and decomposition. To model the annual loss from the charcoal carbon pool, a pool-size based approach is employed in WPsCS Estimator (Eq. 1) [24].

1 $${\rho }_{cha}=\tau +\sigma \times \text{l}\text{n}\left({C}_{cha}\right)$$

  where ${\rho }_{cha}$ is the annual charcoal loss rate (fraction of the pool), $\tau$ is the basic loss rate, $\sigma$ is the pool-size related loss rate, and ${C}_{cha}$ is the carbon pool size of charcoal (kg).

The carbon storage lifetimes vary significantly among the different end-use wood products, from short-term directly disposed wood products such as household paper to long-lasting building materials [25]. To model the annual disposal rate for different wood products, a service-life based approach is used in WPsCS Estimator. This method incorporates the time since production and average service half-life, along with a Chi-squared regression model to estimate the annual disposal rate (Eq. 2) [4, 20]. Therefore, for a given type of end-use wood product made in year *i*, the carbon remaining in in year *j* is accounted for in the product pool that has not reached its service life (Eq. 3) (Integration of Eq.2).

2 $${\rho }_{wp}=\frac{\alpha }{{e}^{\sqrt{2\pi }}}\times {e}^{\frac{-\beta \times {({t}_{w}-\gamma )}^{2}}{\gamma }}$$

3 $${C}_{r}={C}_{w}-{\int }_{0}^{j-i}\frac{\alpha }{{e}^{\sqrt{2\pi }}}\times {e}^{\frac{-\beta \times {({t}_{w}-\delta )}^{2}}{\delta }} {d}_{{t}_{w}}$$

  where ${\rho }_{wp}$ is the annual disposal rate for a type of wood products (fraction of the pool), $\alpha$ and $\beta$ are fitted coefficients (unitless), $\gamma$ is the service half-life (year) of the product type, and ${t}_{w}$ is the time since production (year). In year *j*, ${C}_{r}$ (kg) represents the remaining carbon in the wood products pool that was produced in year *i*, and ${C}_{w}$ (kg) is the total carbon in these wood products produced in year *i*.

A portion of the end-of-life wood materials will be recycled to make new wood products or reused as biofuel, with the remainder disposed to landfills. In WPsCS Estimator, most paper products can be recycled or used as biofuel to generate energy, but wood products for exterior use and household paper are not considered for recycling. Instead, exterior use and household paper wood products are directly disposed to landfills. The recycling rates for wood products are highly dependent on the technology advancement of the wood industry [26]; therefore, to represent the technological advancement in the wood industry influence on recycling rates of disposed wood products, a time-dependent approach is employed in the estimator (Eq. 4) [20]. This recycling rate includes both the carbon reused to make new wood products and as biofuel.

4 $$r=\lambda +\mu \times \text{l}\text{n}\left(k\right)$$

 where *r* is the recycling rate for a type of recyclable wood products, $\lambda$ is the recycling rate in the first year (initial year), $\mu$ represents the effect of industrial advancement on wood products recycling, and *k* is the order of year or the year since the initial year (i.e., 0, 1, 2 … *k*).

The decay rate for each type of waste wood product in the landfill is primarily determined by its physical and chemical characteristics [27]. For example, waste paper has a shorter turnover time than does waste building materials and so are tracked as four separate subpools in WPsCS Estimator. The annual decay rate for each type of waste wood product is modeled by the time since disposition (year) and turnover time (years), along with a log-normal regression model (Eq. 5) [27, 28]. The turnover time is the entire period (number of years) required for the waste wood product in the landfill to be completely decomposed and emitted to the atmosphere.

5 $${\rho }_{lf}=\xi \times \frac{\text{l}\text{n}\left({t}_{l}\right)}{\omega \times \sqrt{2\pi }}$$

 where ${\rho }_{lf}$ is the annual decay rate (fraction of pool) for a type of waste wood products in the landfill, ${t}_{l}$ is the time (year) since disposition (i.e., 0, 1, 2 … $\omega$), $\xi$ is basic decay rate, and $\omega$ is the turnover time (year).

### The estimator interface

**Fig. 2:**
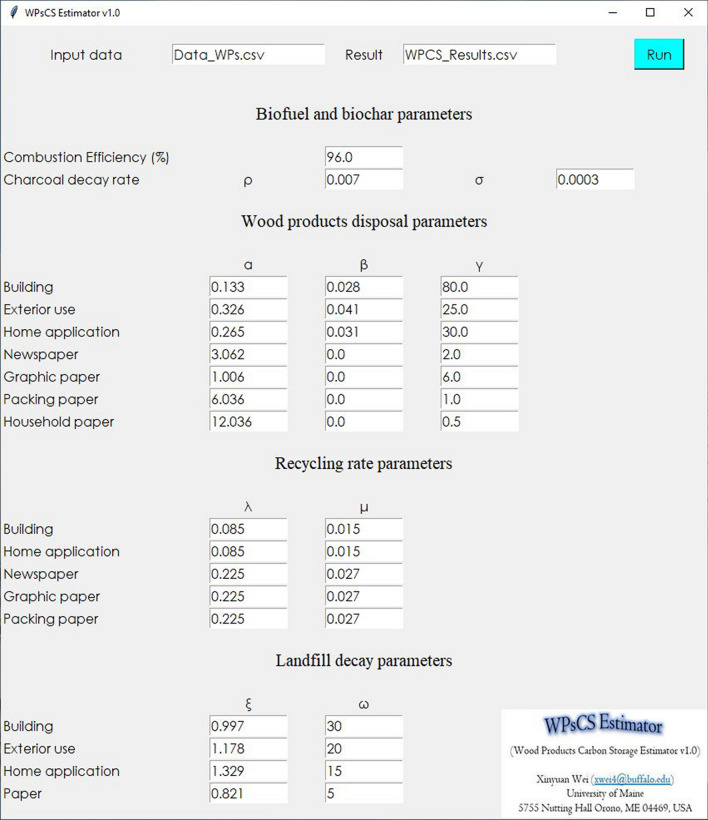
Interface of the Wood Products Carbon Storage Estimator (WPsCS Estimator). The default parameters are used in the two case studies of Maine, USA, and the United States (see "Estimator application" Section). To perform an estimation, see Additional file 1

The WPsCS Estimator is developed using Python programming, and it has a user-friendly interface for its operation (Fig. 2). The data containing the wood product carbon input consists of a comma-separated value (CSV) file including the annual production, consumption, or user-defined system boundary of each wood product (in kg C per year) including non-energy use biochar, building, exterior use, home application, and paper carbon pools. The input wood product data includes products made from harvested timber and recyclable waste wood materials when the system boundary includes the products made from recycled waste wood materials. Parameters including the combustion efficiency of biofuel, charcoal decay rate, disposal rate for each end-use wood product, recycling rate for each recyclable wood product, and decay rate for each type of waste wood material in landfills can be manually calibrated by users (To perform an estimation, see Additional file 1).

### Estimator application

**Fig. 3:**
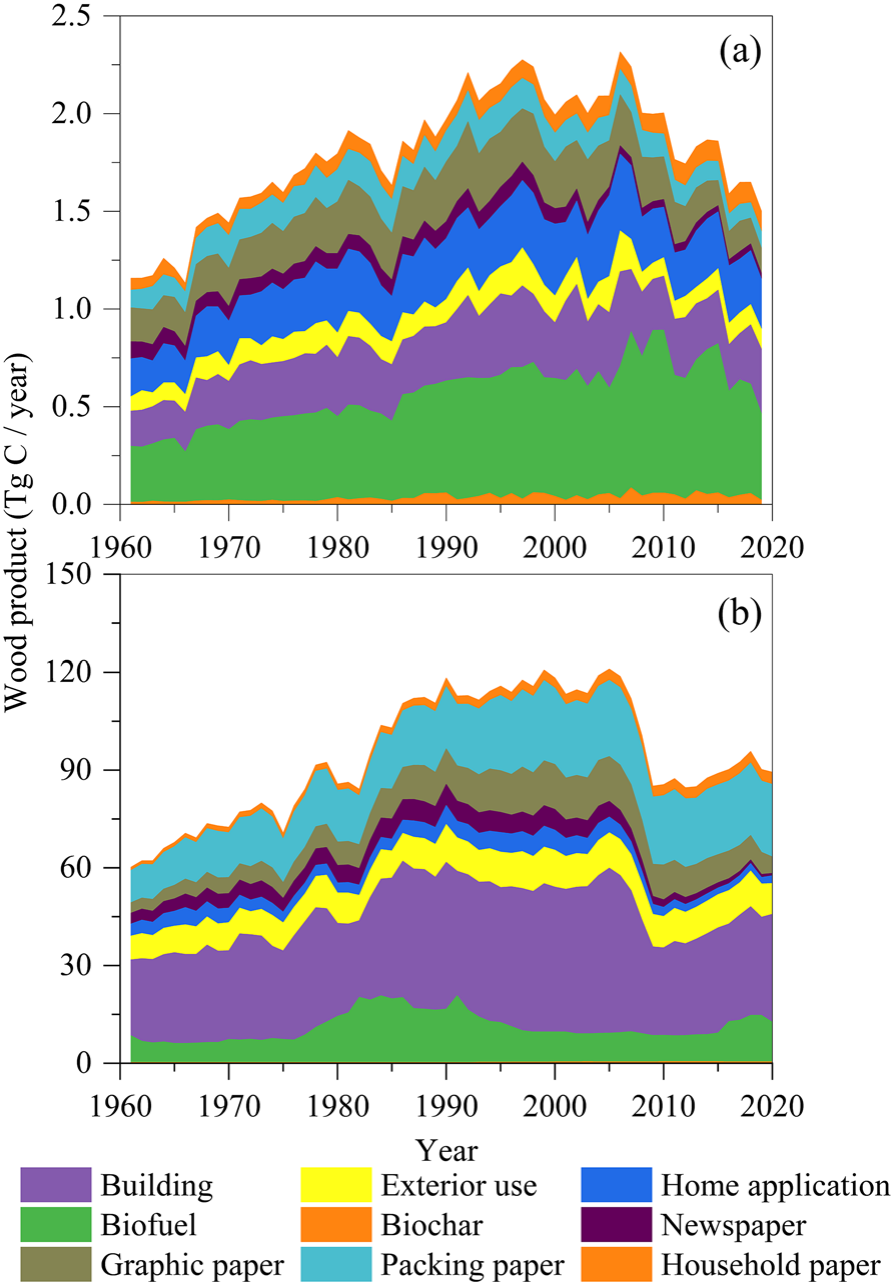
The annual production of wood products made from the timber harvested in Maine, USA during the period of 1961–2019 (**a**), and the annual consumption of wood products in the United States from 1961 to 2020 (**b**)

The WPsCS Estimator was applied to estimate the carbon stored in wood products produced by timber harvested in Maine, USA, over the period of 1961–2019. For this estimation, the production system boundary was employed, meaning that all carbon in wood products harvested in Maine was accounted for regardless of whether it was used in Maine or elsewhere. The timber harvesting data were obtained from the Maine Department of Agriculture Conservation and Forestry (Fig. 3a). To obtain the production of each type of wood products, the allocation method proposed by Li et al. [20] was used. Because this allocation method does not categorize the paper products, the annual fraction of newspaper, graphic paper, packing paper, and household paper of the entire United States provided by the FAOSTAT database [29] was used to allocate paper products.

For a second demonstrative application, the estimator was applied to calculate the carbon storage in wood products consumed in the United States. For this estimation, we used the stock-change system boundary, which estimates the carbon stock in wood products consumed and physically located in the United States, while the wood products exported internationally are not counted. The annual domestic product, as well as the import and export of biofuel, non-energy use biochar, sawlog, structured panel, non-structural panel, paperboard, and paper products in the United States during the period of 1961 to 2020 were obtained from the FAOSTAT database [29]. Therefore, the consumption of each wood product in the United States was calculated as the total of the commercial balance (the difference between import and export) and domestic product (Eq. 6). To allocate these second wood products including sawlog, structured panel, non-structured panel, and paperboard to end-used wood products (Fig. 3b), we applied the consumed solid wood timber products in major end-use markets data in the United States provided by McKeever and Howard [30] and Alderman [31].

6 $${W}_{c}=\left({W}_{i}-{W}_{e}\right)+{W}_{product}$$

 where *W*_*c*_ is the annual consumption of a wood product in the United States, *W*_*i*_ is the imported wood product, *W*_*e*_ is the exported wood product, and *W*_*p*_ is the domestic wood product produced in the United States.

### Estimator parameterization

**Fig. 4:**
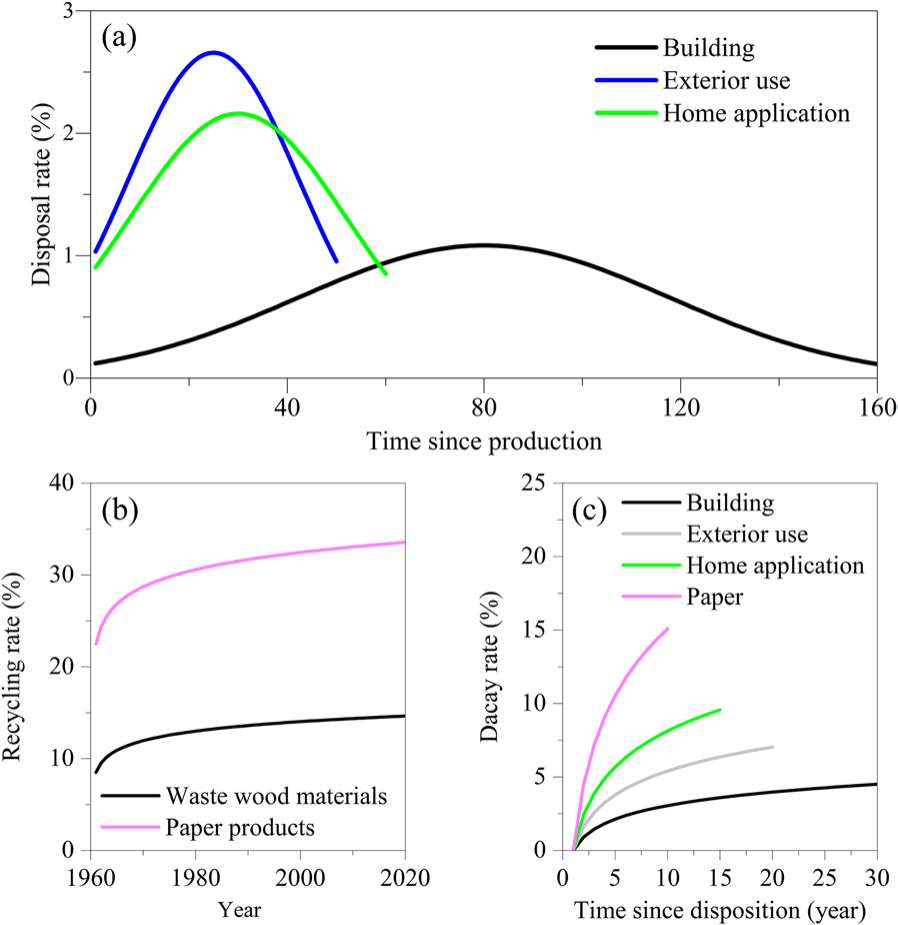
Disposal rates for building, exterior use, and home application wood products (**a**), the recycling rates for solid waste wood materials (building and home application wood products) and paper products (newspaper, graphic paper, and packing paper) (**b**), and decay rates for waste building, exterior use, home application, and paper wood products in landfills (**c**)

Parameters for the WPsCS Estimator can be obtained from expert knowledge and industry surveys or life cycle inventories, but the use of parameters from previous studies is a common practice [32, 33]. To realize the two estimations demonstrated here, we developed a set of parameters including the combustion efficiency, charcoal decay rate, disposal rates for end-use wood products, recycling rates for recyclable disposed wood materials, and decay rates for waste wood products in landfills. The combustion efficiency was obtained from published studies [34–37] and it is an average value for both industrial fuel and residential fuel (Table 1). The charcoal loss rate including decay and reburn rates and related parameters were obtained from global studies conducted by Wei et al. [24] and Landry and Matthews [38] (Table 1). The service half-life for each type of end-use wood product was reviewed from published studies that were conducted in the United States [e.g., 39, 40] (Table 1; Fig. 4a). In the United States, the recycling rate of waste wood materials was obtained from the solid wood products recycling data provided by the United States Environmental Protection Agency (EPA). We suggested that the recycling rate of waste wood materials started from 1961 at a rate of 8.5% and increased to be 14.8% in 2020. The data were used to parameterize the recycling rate regression model (Eq. 4). We employed the same recycling regression model for building and home application wood products (Table 1; Fig. 4b). According to the EPA paper recycling information, the paper recycling rate was estimated as 22.5% in 1961 and significantly increased to a rate of 33.5% in 2020. These rates were used to parametrize the paper recycling regression model, and the parameterized model was then employed for all recyclable paper products including newspaper, graphic paper, and packing paper (Table 1; Fig. 4b). To parameterize the decay regression model for waste wood products in landfills, we drew from the results of several prior studies [i.e., 27, 28] [41–43] (Table 1; Fig. 4c).

**Table 1:** Parameters including the combustion efficiency, charcoal decay rates, disposal rates for end-use wood products, recycling rates for recyclable disposed wood materials, and decay rates for waste wood products used to estimate the carbon stored in wood products made from the timber harvested in Maine, USA, and the carbon stored in the wood products consumed in the United States

Biofuel Biochar	Biofuel and charcoal				
	Combustion efficiency				96%
	Charcoal decay$\left(\tau \right)$				0.007
	Charcoal decay$\left(\sigma \right)$				0.0003
Disposal rate	End-use wood product	α	β		γ
	Building	0.133	0.028		80
	Exterior use	0.326	0.041		25
	Home application	0.265	0.031		30
	Newspaper	3.062	0.0		2
	Graphic paper	1.006	0.0		6
	Packing paper	6.036	0.0		1
	Household paper	12.036	0.0		0.5
Recycle rate	Disposed wood product	λ		µ	
	Building	0.085		0.015	
	Home application	0.085		0.016	
	Newspaper	0.225		0.027	
	Graphic paper	0.225		0.027	
	Packing paper	0.225		0.027	
Landfill decay rate	Waste wood material	ξ		ω	
	Building	0.997		30	
	Exterior use	1.178		20	
	Home application	1.329		15	
	Paper	0.821		5	

## Results

**Fig. 5:**
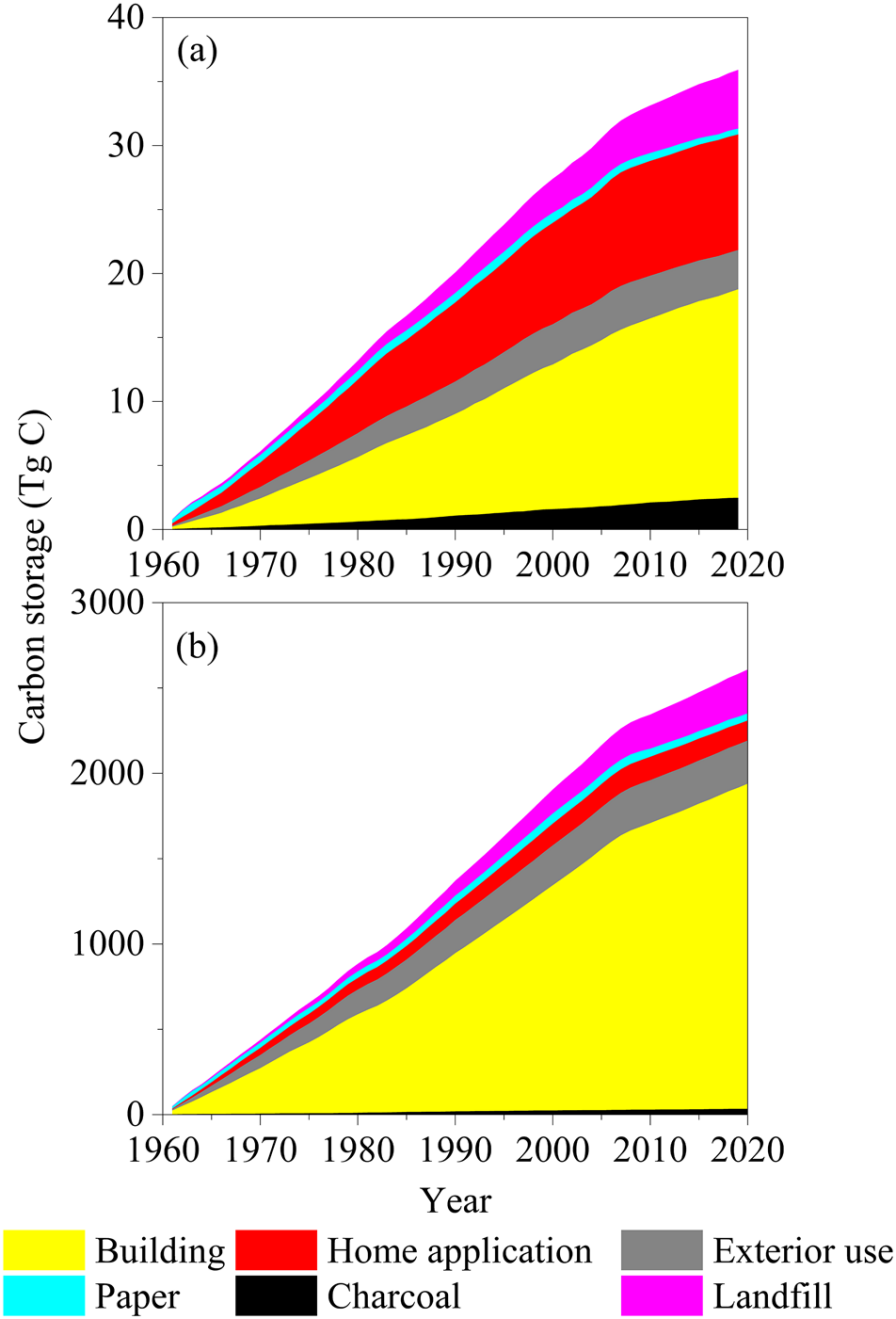
The accumulated carbon storage in wood products made from the timber harvested in Maine, USA, during the period of 1961–2019 (**a**), and the accumulated carbon storage in wood products consumed in the United States during the period of 1961–2020 (**b**)

The carbon stored in wood products made from the timber harvested in Maine, USA, accumulated to 35.89 Tg C from 1961 to 2019, equivalent to an average annual net sink of 0.61 Tg C (Fig. 5a). In 2019, the paper wood products carbon pool had the smallest accumulated size (1.28 Tg C), and the building pool was the largest (16.29 Tg C). Although the average annual production of paper products was 33.31% of the total wood products, due to the fast turnover rate they formed the smallest end-use wood products carbon pool. The home application carbon pool had the second largest size of 9.03 Tg C. Charcoal had the least annual production at an average rate of 0.05 Tg C; however, due to its resistant property charcoal was accumulated to a relatively significant stock representing 6.84% (2.45 Tg C) of the estimated total accumulated carbon storage in Maine-harvested wood products over the period of analysis. In addition, landfill carbon pool stored 4.58 Tg C (12.76%).

The carbon stored in wood products consumed in the United State during the period of 1961–2020 was calculated as 2607 Tg C with an average annual accumulation rate of 43.4 Tg C (Fig. 5b). In 2020, 73.1% (1905.2 Tg C) of the carbon was stored in the building wood products pool, representing the largest end-use pool. Charcoal had the least amount of carbon storage (32.7 Tg C) while paper products had a larger size of 42.4 Tg C. Home application wood products and landfill carbon pool had similar sizes at 252.7 and 256.5 Tg C, respectively, accumulated in the United States over the period of this analysis.

## Discussion

Li et al. [20] reported a carbon storage of 40.3 Tg C in wood products made from the harvested timber in Maine, USA, in the same period, which is higher than our estimates. This is mainly due to the longer service life (15 years) for all paper products employed in their estimation. Skog [44] applied the stock change approach and found that the carbon stock in wood products in the United States increased to 44 Tg C in 2005, while our estimate suggested an increase of 56 Tg C from 1961 to 2019. This is because Skog [44] employed a shorter service life (2 years) for all paper products, did not include the charcoal carbon pool, and applied a faster turnover approach to model the decomposition of waste wood materials in landfills. Zhang et al. [4] reported that the carbon accumulated in end-use wood products in the United States was estimated to be 818 Tg C from 1992 to 2015, which is similar to our result (797 Tg C). Overall, the WPsCS Estimator can successfully account for the carbon stored in wood products. As a part of the lateral carbon export from forest ecosystems [45], accounting for the carbon storage in wood products is required to reconcile the discrepancy between bottom-up estimates of carbon stock change with top-down estimates of land-atmosphere carbon exchange [46, 47]. This estimator can be widely applied to quantify carbon stock changes. The bucking allocation processes that transfers carbon from primary to secondary and ultimately to end-use wood products are omitted in the estimator. Because the production of each type of end-use wood products has significant interannual dynamics, it is a challenge to use a single regression model or a fraction to represent the allocation process over a longer time period [21, 48]. Therefore, the off-the-shelf allocated wood products data is required for the estimator.

The service life for each type of wood product is a critical parameter needed for quantifying the carbon stored in end-use wood products. In this study, parameters obtained from studies conducted in the United States are used to quantify the wood products carbon storage for both Maine and the United States. The service life of each type of wood product varies by region. For example, the service life of wood products used for home application is highly correlated with household income [4, 49], and they have longer service lives in developed countries than developing countries. Therefore, region-specific parameters are essential to obtaining reliable estimates. Ignoring the recycling process may overestimate the carbon inflow rate for the landfill carbon pool; therefore, is essential to include the recycling process in wood products carbon budget estimations. The WPsCS Estimator uses a time-dependent approach to represent the effect of the Industrial Revolution on the waste wood materials recycling. But the processes that use recycled wood products to make new wood products or used as biofuel to generate energy are not modeled in WPsCS Estimator. Thus, the input data should include the wood products made with recycled wood materials or the system boundary should be clarified before organizing the input data.

## Conclusion

The goal for developing the WPsCS Estimator is to efficiently and easily quantify the carbon stored in harvested wood products for a given region over a specific period, which was demonstrated with two illustrative examples. WPsCS Estimator has a user-friendly interface, and all parameters can be easily modified. Because the bucking allocation process is excluded in the estimator, the allocated end-use wood products data is required. This exclusion increases the work to prepare the input data, but it can make the results more reliable. We employed time-dependent methods for the recycling process, which can partially incorporate the influence of technological advancement in the wood industry on waste wood materials recycling. Meanwhile, technological advancement also can extend the service life of wood products used for building and home application [50]. The current simulator uses a constant service life for the entire simulation; therefore, it is weak in representing this effect. Despite these noted limitations, the WPsCS Estimator has broad utility and application for policymakers and practitioners to quantify the impact of wood product processing, consumption, and recycling on local, regional, and global carbon stocks.

## Supplementary Information

**Additional file 1: Text 1.** Description of the WPsCS Estimator. **Table S1.** Parameters include the combustion efficiency, charcoal decay rates, disposal rates for end-use wood products, recycling rates for recyclable disposed wood materials, and decay rates for waste wood products. (These parameters are used for the United States. See the main article to obtain the details for each parameter.)

## Data Availability

The harvesting data of Maine, USA is obtained from Maine Forest Service and Department of Agriculture, Conservation and Forestry (https://digitalmaine.com/for_docs/index.html). The consumed primary wood products of the United Stated is obtained from the Food and Agriculture Organization of the United Nations (https://www.fao.org/faostat/en/#data/FO). The data used to fit recycling rate regression models were obtained from the United States Environmental Protection Agency (EPA) (https://www.epa.gov/facts-and-figures-about-materials-waste-and-recycling/national-overview-facts-and-figures-materials#recycling).

## Abbreviation

WPsCS Estimator: Wood products carbon storage estimator

## Code and Software Availability

The Wood Products Carbon Storage Estimator (WPsCS Estimator) was developed by Python programming and the estimator can be accessed in GitHub (https://github.com/xinyuanwylb19/WPsCS-Estimator).
