# Supplementary material for: A life cycle and product type based estimator for quantifying the carbon stored in wood products
Source: Carbon Balance Manag. 2023 Jan 16;18:1. doi: 10.1186/s13021-022-00220-y (PMC9844030; doi:10.1186/s13021-022-00220-y)
Supplement: Supplementary file 1 — Additional file 1: Text 1. Description of the WPsCS Estimator. Table S1. Parameters include the combustion efficiency, charcoal decay rates, disposal rates for end-use wood products, recycling rates for recyclable disposed wood materials, and decay rates for waste wood products. (These parameters are used for the United States. See the main article to obtain the details for each parameter.) [file 13021_2022_220_MOESM1_ESM.pdf]

Wood Products Carbon Storage Estimator (WPsCS Estimator) Documentation

Xinyuan Wei [1, 2], Jianheng Zhao [2, 3], Daniel Hayes [3], Adam Daigneault [3], He Zhu [4]

[1] Environmental Sciences Division, Oak Ridge National Laboratory, Oak Ridge, TN 37830, USA

[2] Center for Research on Sustainable Forests, University of Maine, Orono, ME 04469, USA

[3] School of Forest Resources, University of Maine, Orono, ME 04469, USA

[4] Institute of Geographic Sciences and Natural Resources Research, Chinese Academy of Sciences, Beijing 100101, China

Contact: Xinyuan Wei (xwei4@buffalo.edu)

Text S1. Description of the WPsCS Estimator.

The WPsCS Estimator is developed by Python programming.

The operating systems required for WPsCS Estimator is Windows 7, 10, 11.

The input data of annual wood products is a comma-separated values (CSV) file including the annual production, consumption, or user-defined system boundary of each wood product broken out by bioenergy, non-energy use biochar, building, exterior use, home application, and paper wood products. Paper products include newspaper, graphic paper, packing paper, and household paper (see the two examples). Note that when the system boundary includes wood products made from recycled waste wood materials, the input wood product data should include products made from harvested timber and recyclable waste wood materials. The unit for each input wood products is **kg C per year**.

The file names of input wood products and output results are provided by the user at the top of the estimator window (Figure 1).

Parameters including the combustion efficiency of biofuel, charcoal decay rate, disposal rate for each end-use wood product, recycling rate for each recyclable wood product, and decay rate for each type of waste wood material in landfills (Figure S1) can be manually calibrated by users (Table 1).

A file named ‘WP_Data’ should be created and placed in the same directory as the estimator, which is used to store the input data and output results.

To start a calculation, click the “Run” button and the calculation will be automatically started. The results are output as a CSV file and listed as the size of each carbon pool for every year.

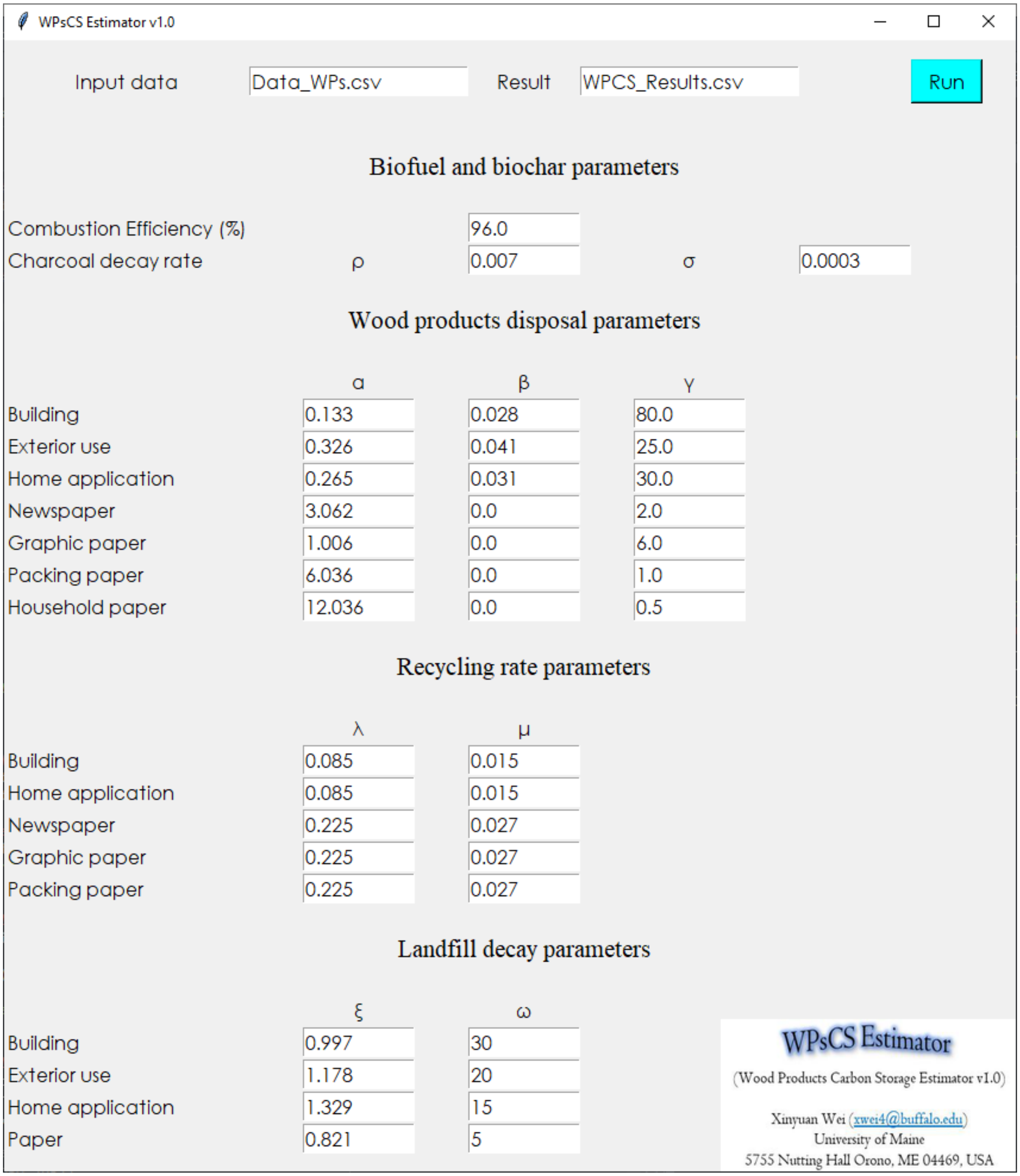

Figure S1. Interface of the Wood Products Carbon Storage Estimator (WPsCS Estimator). The default parameters are used in the two case studies of Maine, USA, and the United States (see examples).

Table S1. Parameters include the combustion efficiency, charcoal decay rates, disposal rates for end-use wood products, recycling rates for recyclable disposed wood materials, and decay rates for waste wood products. (These parameters are used for the United States. See the main article to obtain the details for each parameter.)

Biofuel Biochar	Biofuel and charcoal			
	Combustion efficiency			96%
	Charcoal decay (τ)			0.007
	Charcoal decay (σ)			0.0003
Disposal rate	End-use wood product	α	β	γ
	Building	0.133	0.028	80
	Exterior use	0.326	0.041	25
	Home application	0.265	0.031	30
	Newspaper	3.062	0.0	2
	Graphic paper	1.006	0.0	6
	Packing paper	6.036	0.0	1
	Household paper	12.036	0.0	0.5
Recycle rate	Disposed wood product	λ		μ
	Building	0.085		0.015
	Home application	0.085		0.016
	Newspaper	0.225		0.027
	Graphic paper	0.225		0.027
	Packing paper	0.225		0.027
Landfill decay rate	Waste wood material	ξ		ω
	Building	0.997		30
	Exterior use	1.178		20
	Home application	1.329		15
	Paper	0.821		5

¶ The charcoal loss rate including decay and reburn rates and related parameters were obtained from global studies conducted by Wei et al. (2018) and Landry and Matthews (2017). The service half-life for each type of end-use wood product was reviewed from published studies that were conducted in the United States (e.g., Skog and Nicholson, 2000; Smith et al., 2006). The recycling rate of waste wood materials was obtained from the solid wood products recycling data provided by the United States Environmental Protection Agency (EPA) (https://www.epa.gov/facts-and-figures-about-materials-waste-and-recycling/national-overview-facts-and-figures-materials#recycling).

References

Landry, J.S. and Matthews, H.D., 2017. The global pyrogenic carbon cycle and its impact on the level of atmospheric CO_2 over past and future centuries. Global Change Biology.

Skog, K. E., and Nicholson, G. A. (2000). "Carbon sequestration in wood and paper products," in The impact of climate change on America"s forests: A technical document supporting the 2000 USDA Forest Service RPA Assessment. Gen. Tech. Rep. RMRS-GTR-59, eds L. A. Joyce and R. Birdsey (Fort Collins, CO: U.S. Department of Agriculture, Forest Service), 79–88.

Smith, J.E., Heath, L.S., Skog, K.E. and Birdsey, R.A., 2006. Methods for Calculating Forest Ecosystem and Harvested Carbon with Standard Estimates for Forest Types of the United States General Technical Report (USDA, Forest Service, 2006).

Wei, X., Hayes, D.J., Fraver, S. and Chen, G., 2018. Global Pyrogenic Carbon Production During Recent Decades Has Created the Potential for a Large, Long-Term Sink of Atmospheric CO_2. Journal of Geophysical Research: Biogeosciences, 123(12): 3682-3696.
